# Methods of data collection and definitions of cardiac outcomes in the Rotterdam Study

**DOI:** 10.1007/s10654-012-9668-8

**Published:** 2012-03-03

**Authors:** Maarten J. G. Leening, Maryam Kavousi, Jan Heeringa, Frank J. A. van Rooij, Jolande Verkroost-van Heemst, Jaap W. Deckers, Francesco U. S. Mattace-Raso, Gijsbertus Ziere, Albert Hofman, Bruno H. Ch. Stricker, Jacqueline C. M. Witteman

**Affiliations:** 1 Department of Epidemiology, Erasmus Medical Center, PO Box 2040, 3000 CA Rotterdam, The Netherlands; 2 Department of Cardiology, Erasmus Medical Center, Rotterdam, The Netherlands; 3 Department of Internal Medicine, Erasmus Medical Center, Rotterdam, The Netherlands; 4 Department of Geriatric Medicine, Havenziekenhuis, Rotterdam, The Netherlands; 5 Department of Medical Informatics, Erasmus Medical Center, Rotterdam, The Netherlands; 6 Inspectorate for Health Care, The Hague, The Netherlands

**Keywords:** Cardiovascular disease, Coronary heart disease, Myocardial infarction, Coronary revascularization, Heart failure, Atrial fibrillation, Unrecognized myocardial infarction, Sudden cardiac death, Diagnostic guidelines, Epidemiologic methods, Population-based

## Abstract

The prevalence of cardiovascular diseases is rising. Therefore, adequate risk prediction and identification of its determinants is increasingly important. The Rotterdam Study is a prospective population-based cohort study ongoing since 1990 in the city of Rotterdam, The Netherlands. One of the main targets of the Rotterdam Study is to identify the determinants and prognosis of cardiovascular diseases. Case finding in epidemiological studies is strongly depending on various sources of follow-up and clear outcome definitions. The sources used for collection of data in the Rotterdam Study are diverse and the definitions of outcomes in the Rotterdam Study have changed due to the introduction of novel diagnostics and therapeutic interventions. This article gives the methods for data collection and the up-to-date definitions of the cardiac outcomes based on international guidelines, including the recently adopted cardiovascular disease mortality definitions. In all, detailed description of cardiac outcome definitions enhances the possibility to make comparisons with other studies in the field of cardiovascular research and may increase the strength of collaborations.

## Introduction

Despite major advances in prevention and treatment, the prevalence of cardiovascular diseases (CVD) is rising [1, 2]. Nowadays, the majority of healthy adults will be confronted with some form of CVD during their lifetime and still heart disease is the leading cause of death in the western world, claiming approximately one out of every five lives [2]. Therefore, the continued search for determinants and predictors of occurrence and prognosis of CVD is of paramount importance.

The Rotterdam Study is a prospective population-based cohort study ongoing since 1990 in a suburb of the city of Rotterdam, The Netherlands. The original cohort comprised of 7,983 inhabitants, aged 55 years or over and living in the well-defined Ommoord city district. The participants undergo repeated extensive examinations every 3–4 years at the Rotterdam Study research center, located in the middle of the study area. They are followed for a variety of diseases that are frequent in the general population. At initiation, the study focused on cardiovascular, neurological, ophthalmological, and endocrine diseases. The rationale and design of the Rotterdam Study have been described extensively in EJE two decades ago [3]. However, over the years the original cohort has been extended twice, the scope has been broadened, and the characteristics of repeated examinations have changed. As of December 2008, 14,926 subjects aged 45 years or over comprise the Rotterdam Study cohort. Therefore, its objectives and design have been updated regularly in this journal [4–7]. Parallel to extensions in the design of the Rotterdam Study, medical technology has advanced and clinical presentation of heart disease is evolving.

Within the Rotterdam Study, multiple cardiac outcomes are considered, namely recognized and unrecognized myocardial infarction (MI), myocardial revascularization, coronary heart disease (CHD) mortality, heart failure, atrial fibrillation (AF), and sudden cardiac death (SCD). The sources used for collecting the data are diverse and up until now their corresponding methods and the definitions of various cardiac outcomes in the Rotterdam Study have not been reported combined together in an overview. Furthermore, implementation of novel diagnostics and therapeutic interventions in everyday cardiac care has urged us to change definitions since our earliest reports on prevalence and incidence of MI and CHD [8–10]. Above all, in today’s era of large transatlantic collaborations in epidemiologic research comparability of outcome definitions has gained importance [11, 12]. In this article the methods of data collection and up-to-date definitions of the cardiac outcomes in the Rotterdam Study will be presented.

## Methods of data collection

### Dutch health care system

In order to understand the methods of data collection used in the Rotterdam Study a brief introduction into the Dutch health care system is essential. Primary care, provided by general practitioners (GPs), plays a central role in the health care in The Netherlands. In The Netherlands there are almost 11,000 practicing GPs, who each have had 3 years of specialist training in family medicine. All Dutch inhabitants can register at a single general practice of choice. The GPs act as the gatekeepers to hospital care and must give their approval before patients can get referred to a medical specialist. In doing so, the vast majority of problems presented in primary care are handled by the GPs themselves [13].

For decades, emergency and after-hours care is handled by primary care cooperatives. In order to provide adequate after-hours care, full electronic exchange of patient data is of great importance. Therefore, virtually all GPs in The Netherlands use computer-based GP information systems based on requirements set by the professional organizations of Dutch general practice [Dutch GP Society (NHG) and Nationwide Association of GPs (LVH)]. These computer systems have been designed specifically for use in primary care and consist of a set of specific modules (e.g. medical, electronic communication, prescription, financial). Using the digital systems, all encounters in primary practice are coded using the International Classification of Primary Care (ICPC) [14, 15]. As discussed above, Dutch GPs have a coordinating role in the overall health care utilization process of their patients enlisted. They refer their patients to medical specialists and are reported back on every hospital admission and results from outpatient contacts with medical specialists, preferably using electronic communication [16]. Further details on the structure of the health care system in The Netherlands and the use of electronic medical records have been described in detail [13, 17–20].

With regards to concerns of general accessibility to medical care in The Netherlands, insurance status is not allowed to be considered in referral of patients. Cardiac hospital care, including invasive procedures, is covered by the basic health care insurance plan in The Netherlands, which is obligatory by law [17, 18].

### Assessment of cardiovascular disease status at baseline

Upon entrance in the Rotterdam Study cohort, baseline CVD status of each participant is ascertained in the following way. During a baseline home interview, trained nonmedical interviewers administer a standardized questionnaire to obtain information on medical history (e.g. MI, myocardial revascularization) and health status (e.g. chest discomfort, breathlessness), and labels of current medication are copied (both prescription and over-the-counter usage). Drug use is coded according to the Anatomical Therapeutic Chemical (ATC) classification index [21]. Questions on indication of cardiovascular medication and breathlessness were lacking at the start of the Rotterdam Study, but have subsequently been added. Consequently, these questions were asked in most (70%) of the participants at baseline of the original Rotterdam Study cohort. After the interview, the participants are invited to visit the research center where they undergo a physical examination in some detail by one of the study physicians and various tests are performed [e.g. resting electrocardiogram (ECG), echocardiography]. In addition to these examinations, information on prevalent disease status is obtained by accessing data from the Nationwide Medical Registry (LMR, sourced by Dutch Hospital Data, Utrecht, The Netherlands). This is a national registration on all primary and secondary hospital discharge diagnoses of Dutch inhabitants, with linkage on the basis of zip code, date of birth, gender, and GP. Records from this registry are linked to the study database. For potential events identified in this registry, copies of hospital discharge letters and ECGs are requested. Most importantly, clinical information on prevalent CVD status is obtained from the GPs for each participant: the entire medical records of the GPs are hand screened at the GPs’ office by trained research assistants. Using the aforementioned sources (interview, examination at the research center, Nationwide Medical Registry, and full screening of GPs’ records), disease status at baseline is available for all participants of the Rotterdam Study. An overview of the sources used for ascertaining disease status at baseline is presented in Table 1.

**Table 1:** Sources of data in the Rotterdam Study

Source	Data obtained on disease status at study baseline	Data obtained on occurrence of outcomes during follow-up
Regular checks of medical records at the GPs’ office	Full medical history	Intercurrent medical history
	Hospital discharge letters	Intercurrent hospital discharge letters
	Reports on outpatient contacts with medical specialists	Intercurrent reports on outpatient contacts with medical specialists
	Previous ECGs	Intercurrent ECGs
		Cause and circumstances of death
Continuous linkage of the study database with GPs’ digital files	NA	ICPC codes of all diagnoses made Date of death
Home interviews	Medical history	Intercurrent medical history
	Current health status	Current health status
	Current medication use	Current medication use
Research center visits	Resting ECG	Resting ECG
	Physical examination	
Pharmacy prescription records	Current medication use	Continous monitoring of all prescriptions filled
Nationwide Medical Registry (LMR)	History of hospital discharge diagnoses for any outcome of interest	Intercurrent hospitalization with AF or atrial flutter
Municipality records	NA	Date and place of death
Hospitals	Hospital discharge letters	Hospital discharge letters
	Previous ECGs	Intercurrent ECGs

*GP* general practitioner, *ECG* electrocardiogram, *NA* not applicable, *ICPC* International Classification of Primary Care, *AF* atrial fibrillation

### Clinical follow-up

Follow-up starts after the baseline home interview of each individual participant. Data on clinical cardiovascular outcomes are collected continuously through an automated follow-up system. The follow-up system involves automated digital linkage of the study database to digital files from GPs in the study area. On a weekly basis, all ICPC codes of diagnoses of interest made by the GPs and medical specialists in study participants are entered to the Rotterdam Study database. Moreover, the entire medical record of each participant living in the research area is checked by hand on a regular basis at the GPs’ office by trained research assistants for diagnoses of interest. This is the primary source of information on CVD events, since all letters of medical specialists, discharge reports in case of hospitalization, and ECGs are copied by the research assistants. Subsequently, all the collected information is compared to the ICPC codes entered to the study database for each individual participant. This is done in order to make sure no clinical information on potential events is missed out. Additional information is obtained from the hospitals in case the automated follow-up system or medical records contain insufficient information. Medical records of the participants under the care of nursing home physicians or GPs working outside the study area are checked annually for potential events. Furthermore, before every repeat examination the participants are interviewed on the occurrence of cardiac events since their last visit to the research center.

With respect to the vital status of all participants, information is obtained on a weekly basis from the central registry of the municipality in Rotterdam and through the digital linkage with GPs working in the study area. For participants living outside the research area, the GPs are the primary source of information, complemented by the municipality records in the place of residence. After notification, cause and circumstances of death are established by requesting information from the medical records of the GPs or nursing home physicians.

As of January 1991 onwards all drug prescriptions dispensed to participants by seven fully automated pharmacies in the study area are routinely stored in the database. At baseline, nearly all (99.7%) participants were registered at one of these pharmacies. This data consists of information on the date of delivery, the total amount of drug units per prescription, the prescribed daily number of units, product name of the drugs, and the ATC-code [21, 22].

Table 1 provides an overview of the sources used for obtaining information on the occurrence of cardiac outcomes during follow-up.

### Electrocardiography

At baseline and at each follow-up visit to the research center, every participant has a 10 s 12-lead resting ECG (on average 8-10 beats) recorded using an ACTA Gnosis IV ECG recorder (Esaote Biomedica, Florence, Italy) at a sampling frequency of 500 Hz and stored digitally. All ECGs are processed by the standardized Modular ECG Analysis System (MEANS) to obtain ECG measurements and interpretation. The ECGs are analyzed off-line using MEANS. The MEANS program has been evaluated extensively and determines common onsets and offsets for all 12 leads together on one representative averaged beat, with the use of template matching techniques [23–27].

### Ethics approval

The Rotterdam Study has been approved by the institutional review board (Medical Ethics Committee) of the Erasmus Medical Center and by the review board of The Netherlands Ministry of Health, Welfare, and Sports. The approval has been renewed every 5 years.

### Informed consent

Participants provided written informed consent to participate in the study and to obtain information from their treating physicians, separately. The latter includes permission to obtain information from the GP, medical specialists, and pharmacists.

### Event adjudication

For each outcome two cardiovascular research physicians independently classify information on occurrence, certainty, and date of onset of all data collected on potential events according to the corresponding definitions below. Cases on which the research physicians disagree are discussed in order to reach consensus in a separate session. Afterwards, a panel of medical specialists in CVD reviews potential events for each diagnosis separately. This panel consists of a cardiologist (J.W.D.), two geriatricians (F.U.S.M.-R. and G.Z.), and a GP experienced in cardiac disease (J.H.). The medical specialist’s judgment is considered decisive. The research physicians and the medical specialists base their decisions on the same data. This procedure is similar for both prevalent and incident outcomes.

## Definitions of cardiac outcomes

Within the Rotterdam Study eight highly prevalent cardiac outcomes are considered subdivided into three categories, namely CHD, heart failure, and cardiac arrhythmias (Table 2).

**Table 2:** Cardiac outcomes in the Rotterdam Study

Categories	Underlying outcomes
Coronary heart disease	MI
	Unrecognized MI
	Myocardial revascularization
	CHD mortality
	Overall CHD
Heart failure	Heart failure
Cardiac arrhythmia	Atrial fibrillation and atrial flutter
	Sudden cardiac death

*MI* myocardial infarction, *CHD* coronary heart disease

## Coronary heart disease

### Myocardial infarction

The triad of chest pain, ECG abnormalities, and rise of cardiac enzymes has been a generally accepted definition of acute MI for many years. However, during the past decades development of more sensitive and specific blood markers (e.g. creatinin kinase MB, troponins) and enhanced imaging techniques allow for detection of smaller MIs. Widespread introduction of troponin testing in The Netherlands happened around the turn of the century and took several years to be fully implemented in the hospitals in the research area [28]. This has had implications for adjudication of MIs in the Rotterdam Study. Accordingly, clinical practice, as well as epidemiological research required a more precise definition of MI [29].

Methods on follow-up and event adjudication of prevalent and incident MI for the Rotterdam Study have been described previously in brief [30]. The diagnosis of MI is classified as definite, probable, possible or unlikely. Definite MI is defined as pathology findings of an acute MI within 28 days of death, or a rise/fall in cardiac biomarkers and/or objective indicative ECG changes, and preferably the presence of symptoms or signs (e.g. cardiac pain, cardiogenic shock). Also, for definite MI, the diagnosis has to have been made by a medical specialist, preferably a cardiologist or an internist. If the MI was diagnosed by a GP or a nursing home physician it is classified as probable. MI is classified as possible when one of the criteria for probable or definite MI cannot be met. MI is considered unlikely if symptoms or signs are present, but objective evidence showing myocardial necrosis is lacking. Accordingly, diagnoses of unstable angina, acute coronary syndromes, and invasive procedure related ischemia are also considered as MI events whenever they are accompanied by a significant rise in cardiac biomarkers. Thereby, the current definition of MI in the Rotterdam Study includes the clinical type 1, 2, 4a, 4b, and type 5 MI as defined in the endorsed universal definition of MI [29]. In accordance with the international epidemiological CHD case definitions, only definite and probable cases are included in the Rotterdam Study definition, unless otherwise noted [31]. For participants of the original Rotterdam Study cohort, the presence of MI at baseline is based on verification of either self-reported MI or ECG abnormalities indicative of prior MI. In subsequent cohorts, the medical records of all participants are screened for prevalent MI, regardless of their self-reported history or ECG abnormalities. The presence of MI during follow-up is based on clinical information from the medical records. The date of incident MI is defined as the day of the first occurrence of symptoms suggestive of MI.

### Unrecognized myocardial infarction

Unrecognized MI, although prevalent, is not always considered as an outcome in epidemiologic studies on CHD, since determining an exact date of occurrence of the MI is impossible by definition [30]. Therefore, unrecognized MI is not included as an outcome in studies on the occurrence of CHD in the Rotterdam Study, unless otherwise noted. However, separate studies within the framework of the Rotterdam Study have been conducted on the prognosis of this type of presentation of CHD [32–34].

Methods on definition of prevalent and incident unrecognized MI within the Rotterdam Study have been summarized previously [30, 34]. This definition is in accordance with the criteria for ‘prior MI’ (type 3) defined by the international Task Force for the Redefinition of MI, as follows in detail [29]. At baseline of the Rotterdam Study all participants were asked whether they had ever experienced a heart attack and who established the diagnosis. Afterwards, an ECG was obtained and analyzed using MEANS as described above. To determine MI, MEANS uses a comprehensive set of criteria that partly derive from The Minnesota Code [35, 36]. Pathological Q-waves are central in the diagnosis of MI using MEANS, next to auxiliary criteria, such as QR-ratio and R-wave progression. A cardiologist with expertise in electrocardiography, whose judgment was considered final, reviewed all cases that were classified by MEANS as possible, probable, or definite MI. At baseline of the Rotterdam Study, unrecognized MI was considered to be present in all participants with confirmed ECG characteristics matching a MI, but without documented history or self-reported MI. An incident unrecognized MI is considered to have occurred if there is confirmed electrocardiographic evidence of MI on follow-up examinations at the research center, given the absence of an incident clinically recognized MI at baseline or during follow-up. The unrecognized MI is considered to have occurred in the middle of the time interval between the examination at which the unrecognized MI is detected and the examination before that.

### Myocardial revascularization

Invasive myocardial revascularization is an established treatment for acute MI, relief of unstable angina, and medically intractable stable angina. Furthermore, patients with symptomatic or asymptomatic severe coronary artery disease benefit from myocardial revascularization by improving survival [37]. Especially during the past decade, a great number of novel and hybrid cardiac interventions, and other transcatheter interventions have been introduced [37, 38].

Within the Rotterdam Study data is collected on incident coronary artery bypass grafting (CABG) and percutaneous coronary interventions (PCIs) for atherosclerotic CHD, separately. For PCI, previously termed percutaneous transluminal coronary angioplasty, the following interventions are considered: coronary stenting, coronary balloon angioplasty, coronary recanalization, intracoronary thrombosuction, and (although very rare) intracoronary laser and brachytherapy. CABG and PCI are also adjudicated for combined cardiopulmonary surgery and other combined or hybrid cardiac procedures [37, 38]. Any attempt of revascularization is adjudicated, regardless of success, given the indication is still present at the time of the attempt. For participants of the original Rotterdam Study cohort, the presence of myocardial revascularization at baseline is based on self-reported CABG or PCI, verified by clinical data from the medical records. In subsequent cohorts, the medical records of all participants are screened for prevalent myocardial revascularization procedures, regardless of their self-reported history. The presence of CABG and PCI during follow-up is based on clinical information from the medical records. The date of incident myocardial revascularization is obtained from the hospital discharge letters.

As mentioned before, myocardial revascularization procedures are available to everyone in The Netherlands, regardless of insurance status. Myocardial revascularization is fully covered by the basic health care insurance in The Netherlands, which is obligatory by law [17, 18].

### Coronary heart disease mortality

Fatal CHD is often an unheralded presentation of presymptomatic coronary artery disease and is mainly attributed to sudden death, ischemic heart failure, and sequelae of a MI [39]. Originally, the CHD mortality definitions in the Rotterdam Study have been based on the International Classification of Diseases, 10th revision (ICD-10) codings [40, 41]. Recently, a classification used in other large cohort studies with specific focus on CVD has been adopted in order to improve the quality of the outcome data and enhance comparability with other epidemiological studies. This system is a marginally adapted classification applied by both the Cardiovascular Health Study and the Atherosclerosis Risk in Communities Study [42–45]. From 2003 onward, this classification has served as a basis for the endorsed international case definition for out-of-hospital CHD mortality in epidemiologic studies [31].

As a first step, all deaths (both cardiovascular and noncardiovascular) in the Rotterdam Study are adjudicated based on ICD-10 codes. Subsequently, all available clinical information for each potential new fatal CHD and CVD case is reviewed by the research physicians in order to ascertain the underlying cause of death and adjudicate a CHD or CVD mortality category. The underlying definitions for the CHD and CVD mortality categories are presented in Table 3. CVD mortality is subdivided into the following hierarchical categories: CHD (definite fatal MI, definite fatal CHD, and possible fatal CHD), nontraumatic cerebrovascular disease, other atherosclerotic disease, and other CVD. Within the Rotterdam Study, none of the deaths are classified as due to heart failure. The classification system used in the Rotterdam Study focuses on the underlying etiology, rather than the mode of death: i.e. participants dying with decompensated heart failure are mostly classified as deaths being from CHD or valvular heart disease. In rare cases where no possible underlying etiology of heart failure can be established from the medical records, these deaths are classified as being from other CVD. The date of death is established from the medical records or municipality records.

**Table 3:** Rotterdam Study cardiovascular mortality classification and definitions for underlying cause of death

Mortality categories (hierarchical)	Underlying cause of death	
1. Coronary heart disease	Definite fatal MI	No known nonatherosclerotic cause, and definite MI within 28 days of death
	Definite fatal CHD	No known nonatherosclerotic cause, and at least one of the following: cardiac pain within 72 h of death or a history of ischemic heart disease in the absence of significant valvular heart disease or nonischemic cardiomyopathy
	Possible fatal CHD	No known nonatherosclerotic cause, and mode of death consistent with CHD in the absence of significant valvular heart disease or nonischemic cardiomyopathy
2. Cerebrovascular disease		Nontraumatic intracerebral haemorrhage or infarction
3. Other atherosclerotic disease		Atherosclerotic disease other than CHD or cerebrovascular disease (including ruptured abdominal aortic aneurysm, peripheral vascular disease, and visceral vascular disease)
4. Other cardiovascular disease		CVD other than 1–3 (including valvular heart disease, nonischemic cardiomyopathy, endocarditis, hypertensive renal disease, pulmonary embolism, ruptured thoracic aortic aneurysm, and complications from cardiovascular interventions other than 1–3)
5. Noncardiovascular disease		All other causes of death other than 1–4 (including natural, due to trauma, suicide, and death of unknown or uncertain cause)

*MI* myocardial infarction, *CHD* coronary heart disease, *CVD* cardiovascular disease

### Coronary heart disease

The many forms of presentation of CHD make up for many possibilities of combining these into an overall disease outcome. The definition of combined CHD outcomes may depend on the research question at hand.

Within the Rotterdam Study two different combined outcomes have been used as described previously [40]. First, ‘total CHD’ is defined as a combined outcome of myocardial revascularization (as a proxy for significant coronary artery disease), MI (fatal and nonfatal), and fatal CHD. Second, ‘hard CHD’ is defined as MI (fatal and nonfatal) and fatal CHD. Heart failure morbidity and unrecognized MI are not part of the combined CHD definitions, unless otherwise noted.

## Heart failure

The presentation and etiology of heart failure is heterogeneous [46]. Strict case definition and diagnostic criteria for follow-up studies are therefore of utmost importance.

Methods on event adjudication of prevalent and incident heart failure for the Rotterdam Study have been described previously [47, 48]. The diagnosis of heart failure is classified as definite, probable, possible or unlikely. Definite heart failure is defined as a combination of the presence of typical symptoms or signs of heart failure, such as breathlessness at rest or during exertion, ankle edema, and pulmonary crepitations, confirmed by objective evidence of cardiac dysfunction (chest X-ray, echocardiography). This definition is in accordance with the criteria of the European Society of Cardiology (ESC) [46]. Also, for definite heart failure, the diagnosis has to have been made by a medical specialist, preferably a cardiologist or an internist. Heart failure is classified as probable when at least two typical symptoms suggestive of heart failure are present, and at least one of the following: history of CVD (e.g., MI, valvular heart disease, hypertension), positive response to initiated treatment for heart failure, or objective evidence of cardiac dysfunction, while symptoms cannot be attributed to another underlying disease, such as chronic obstructive pulmonary disease. Heart failure is classified as possible when one of the criteria for probable heart failure cannot be met. For both probable and possible heart failure, a diagnosis of a GP or a nursing home physician suffices. Heart failure is considered unlikely if symptoms or signs are present, but when objective evidence fails to show cardiac dysfunction, and if symptoms or signs can be attributed to another underlying disease. In accordance with the ESC guidelines, only definite and probable cases are used in the Rotterdam Study definition [46]. Inclusion of probable heart failure depends on the research question at hand and is detailed in the methods of the corresponding analyses. A participant is not considered as having heart failure, if heart failure occurs directly postoperative after cardiac surgery. For participants of the original Rotterdam Study cohort, the presence of heart failure at baseline is based on clinical information from the medical records for all participants and by using a validated score, similar to the definition of heart failure by the ESC [46, 47, 49]. In subsequent cohorts, the medical records of all participants are screened for prevalent heart failure. The presence of heart failure during follow-up is based on clinical information from the medical records. The date of incident heart failure is defined as the date of the first occurrence of symptoms suggestive of heart failure, obtained from the medical records, or the day of receipt of a first prescription for a loop diuretic or an angiotensin-converting enzyme inhibitor, whichever comes first.

## Cardiac arrhythmia

### Atrial fibrillation and atrial flutter

AF and atrial flutter are the most common sustained cardiac arrhythmia and are well known risk factors for stroke and mortality [50]. Within the Rotterdam Study both AF and atrial flutter are one outcome and referred to as AF, given the likewise natural course [51].

Methods on follow-up and event adjudication of prevalent and incident AF for the Rotterdam Study have been described previously [50]. In accordance with the ESC guidelines, an ECG that verifies the diagnosis for all potential cases of AF is required [52]. A participant is not considered as having AF, if AF occurs during the process of dying and is not the cause of death, or if transient AF occurs during a MI or directly postoperative after cardiopulmonary surgery. The presence of AF at baseline is based on clinical information from the medical records for all participants of the Rotterdam Study. Additionally, at baseline a resting ECG is obtained using the aforementioned methods and analyses software (MEANS). Notably, MEANS is characterized by a high sensitivity (96.6%) and a high specificity (99.5%) in coding arrhythmias [26]. To verify the diagnosis of AF, all ECGs with a diagnosis of AF or atrial flutter or any other rhythm disorder are recoded independently by two research physicians who are blinded to the MEANS diagnosis. The judgment of a cardiologist is asked and taken as decisive in case of persistent disagreement. The presence of AF during follow-up is based on ECG evidence from the medical records. Furthermore, cases of newly diagnosed AF are obtained during the follow-up examinations at the research center and by accessing the hospital discharge diagnoses data from the Nationwide Medical Registry. The date of incident AF is defined as the date of the first occurrence of symptoms suggestive of AF with subsequent ECG verification, obtained from the medical records. When diagnosed at the research center and no other information on a diagnosis of AF is available from either the GPs’ files and/or the Nationwide Medical Registry, the date of onset of AF is defined as the midpoint of the time interval between examination at which AF is detected and the previous examination at the research center.

### Sudden cardiac death

The term SCD is commonly used for a mode of cardiac death. The clinical presentation of sudden cardiac death is frequently used as a surrogate implying that a specific mechanism is involved. The underlying etiology can be diverse, but most often results from tachyarrhythmia or mechanical complications of MI [53]. SCD is an outcome of special interest in studies on genetics, certain ECG parameters, and pharmacological adverse effects on the heart [54–56].

During the past century there has been some debate on the definition of this clinical presentation of heart disease, however Meyerburg’s definition has been accepted and endorsed widely: “A natural death due to cardiac causes, heralded by abrupt loss of consciousness, within 1 h after the onset of acute symptoms or an unwitnessed, unexpected death of someone seen in a stable medical condition less than 24 h previously with no evidence of a non-cardiac cause” [53, 57].

Within the Rotterdam Study the methods of adjudicating SCD are based on the definition supported by the ESC and have been described previously [53, 55]. All available information from GPs and a copy of the medical records are used to assess if the death can be classified as a SCD using the aforementioned definition proposed by Meyerburg [53, 57]. First, potential cases are subdivided on the basis whether the death is witnessed. If death is witnessed and occurs within one hour after the start of symptoms (if present) it is assumed to be a SCD, without additional review of the medical records for a medical history of CVD. In case of an unwitnessed death, evidence of underlying cardiac or noncardiac causes is searched for. Inclusion of unwitnessed SCD in the Rotterdam Study definition depends on the research question at hand. The date of death is established from the medical records or municipality records.

## Discussion

### Comparability

The necessity of research on the etiology and prognosis of heart disease has been obvious for many decades. This has resulted in countless epidemiological studies with a focus on CHD or CVD at large, which is represented by the recent cardiovascular research presented in EJE [58–74]. However, multiple, and sometimes inconsistent, definitions of cardiac outcomes are in use. For instance, the inclusion of stable or unstable angina pectoris, coronary revascularization procedures, and specific subtypes of CHD mortality varies greatly in overall CHD outcome definitions. This may influence the conclusions drawn and impede the overall comparability of studies on CHD.

A substantial proportion of the CHD mortality occurs out-of-hospital [39]. Classification of out-of-hospital death is often based on limited information, due to its sudden onset or unwitnessed occurrence. Therefore, a clear coding system is of key importance. Various classifications by the World Health Organization (WHO), using data from death certificates, or self developed CHD mortality definitions are used in epidemiological and clinical research. Within the Rotterdam Study, ICD-10 has been the classification of choice during the past decade for fatal and nonfatal events [40, 41]. However, the ICD-10 causes confusion when coding mortality. ICD-10 contains codes for both underlying disease (cause of death), as well as mechanisms and circumstances of dying (mode of death). For instance, cardiac arrest (code I46), heart failure (code I50), sudden death (code R96), and unattended death (code R98) could very well be attributed to CHD as to other conditions, depending on ones individual medical history. More recently, in order to avoid confusion and enhance comparability in our multiple large transatlantic collaborations with other epidemiologic studies, a classification used by other large cardiovascular cohort studies has been adopted by the Rotterdam Study [11, 12, 75]. As mentioned before, this classification has been proposed as the international standard for epidemiological research [31]. As a consequence, harmonization of the outcome definitions used in our large epidemiologic collaborations will strengthen the consistency of future results. Furthermore, the categorization of the events as such helps to avoid inaccuracy in the immediate cause of death reporting, complicated by the presence of comorbid conditions, particularly in the elderly [43]. Therewith, this classification allows for more accurate adjudication of the cause of death by underlying etiology and result in less misclassification.

### Variability

Insight into outcome definitions does not only facilitate comparability between studies, it may also explain variability between or even within studies. Reported incidences of CVD vary over different geographic areas and may reflect differences in presence of risk factors, active treatment, and differences in CVD susceptibility among populations. However, the differences may also be a result of differences in coding systems used or differences in clinical practice of adjudication of events [76]. In a recent report on the WHO Burden of Disease Program the incidence of fatal ischemic heart disease varied greatly. For instance, incidence of fatal ischemic heart disease was reported to be 54 per 100,000 in The Netherlands, and 98 per 100,000 in the United States, accounting for 11.3% and 17.9% of the total mortality, respectively. Besides transatlantic differences, great dissimilarities among neighboring nations in Western Europe were observed. Incidences of fatal ischemic heart disease varied from 38 per 100,000 in France to 90 per 100,000 in both Germany and the United Kingdom [77]. Despite the fact that all countries applied the same WHO coding system for adjudication of the causes of death, such differences in incidences are unlikely to be fully explained just by variation in presence and management of cardiovascular risk factors. The precise cause of this variability remains uncertain.

Variability in incidences is also known to occur within the same study. It is well known that calendar time is a cause of variability in observed incidences of heart disease in a single study population due to changes in prevalence or treatment of risk factors, or introduction of novel sensitive diagnostics over time (e.g. creatinin kinase MB, troponins) [78]. Furthermore, researchers may decide on including outcomes of various certainties (e.g. definite, probable). Depending on the research question at hand, more or less sensitive criteria may be applied in different analyses. Next, one should also be aware that a study population is of higher average health status at baseline of a study or shortly after an active repeat research center visit [79]. After all, those who attend in a visit to a research center are necessarily healthy enough to undergo the examinations.

### Quality control

In The Netherlands, many studies on incidence and prognosis of heart disease rely on data provided by the Nationwide Medical Registry. This registry includes all discharge diagnoses for every hospital admission in The Netherlands and has shown a good sensitivity and good positive predictive value for acute CHD diagnoses, but less for chronic conditions such as heart failure [80]. Within the Rotterdam Study, a validation study for evaluating the clinical follow-up event registration of incident MI was performed. This was done by obtaining data on hospital discharge diagnoses in Rotterdam Study participants from the Nationwide Medical Registry. A total of 100 discharge diagnoses of MI were obtained from the registry. In 59 instances MI was the primary discharge diagnosis, and in 41 instances MI was mentioned as a secondary discharge diagnosis. These 100 hospitalized MIs were compared to incident events observed through our clinical follow-up system and this showed that none of the primary diagnoses were missed and only two of the secondary discharge diagnoses were not detected, resulting in a 98% case finding of hospitalized MIs in our study population.

### Strengths and limitations

Within the Rotterdam Study we have over two decades of experience in data collection. It is known that use of various sources for data collection is needed to achieve complete follow-up in large epidemiologic studies [81]. Within the Rotterdam Study, multiple sources for potential events are consulted, namely the linkage of the medical records and pharmacy data to the study database, regular screening of medical records at the GPs’ office, follow-up interviews and examination at the research center, and consultation of the central registry of the local municipality (Table 1). The Rotterdam Study thereby has a virtually complete follow-up with respect to vital status: using the sources described above, exactly 22 years after start of the study, less than 1.8% of the participants have been lost to follow-up. This is predominantly due to emigration.

At initiation of the Rotterdam Study most GPs in the research area were already using standardized digital patient records, resulting in over 85% of the enrolled participants having their medical record digitally linked to the study database [20]. Still, 22 years after initiation of the study, the great majority (79%) of all participants alive are enlisted with a GP with linkage to the automated follow-up system. This results in high quality documentation. Furthermore, the GPs in the research area have a low threshold to refer patients for community based laboratory testing and (exercise) ECGs. However, this does not fully apply to the participants living in nursing homes. Predominantly, the oldest of old and diseased participants are less likely to undergo diagnostic tests (e.g. ECG, echocardiography, cardiac biomarker testing), or to get referred to a medical specialist in comparison to elderly in other European countries [82]. This is usually due to lack of diagnostic accuracy of physical examination and reduced mobility of the nursing home residents [83]. Moreover, clinical benefit is uncertain and care for other comorbid conditions (such as Parkinson’s disease or advanced dementia) is considered to take priority over performing diagnostic procedures outside the nursing home [84, 85]. In all, this may result in missing nonhospitalized nonfatal events in nursing home residents.

Although nowadays the typical cardiac patient is of old age, elderly persons are still highly underrepresented in cardiovascular research [86, 87]. The Rotterdam Study has no upper age limit and can thereby study the determinants and outcomes of heart disease in older participants. This implies challenges in adjudication of diagnosis of especially chronic diseases (e.g. heart failure), which are associated with a wide range of comorbid conditions [88, 89]. Symptoms of other common disease in older individuals, such as chronic obstructive pulmonary disease and chronic venous insufficiency, can be easily misattributed to the failing heart. Strict case definitions are therefore insurmountable in order to prevent misadjudication, however this may result in missing some cases where limited information is available.

## Conclusion

The need for studying occurrence and prognosis of heart disease is obvious. Case finding in epidemiological studies is strongly depending on the availability of various sources of clinical follow-up and clear outcome definitions. The presentation of the up-to-date definitions of cardiac outcomes in epidemiologic studies will result in enhanced possibilities to compare results with other studies in the field of cardiovascular research and may increase the strength of future collaborations.
